# Myelosuppression Rates with Administration of Nafcillin with and without Rifampin in Pediatric Patients

**DOI:** 10.3390/pediatric14020036

**Published:** 2022-06-08

**Authors:** Jolly Kuriakose, Michelle Kaplansky, Caroline M. Sierra

**Affiliations:** 1 Loma Linda University Health, Loma Linda, CA 92350, USA; jkuriakos@presby.edu; 2 School of Pharmacy, Loma Linda University, Loma Linda, CA 92350, USA; mkaplansky@students.llu.edu

**Keywords:** neutropenia, thrombocytopenia, anemia, nafcillin, rifampin, pediatrics

## Abstract

Myelosuppression, a potential adverse reaction of nafcillin and rifampin, is rarely documented in pediatric populations. The objective of this study is to describe the incidence of myelosuppression in pediatric patients receiving nafcillin or a combination of nafcillin and rifampin therapy. This retrospective chart review identified patients who received nafcillin alone or in combination with rifampin. The primary endpoint was the incidence of myelosuppression as a composite outcome. The secondary endpoints were the incidence of thrombocytopenia, anemia, and neutropenia individually. Of 199 patients in this study, 98 received nafcillin alone. There was no difference in the rates of myelosuppression between patients receiving nafcillin alone or in combination with rifampin (*p* = 0.0763), and the use of combination therapy did not affect the development of neutropenia (*p* = 0.2764) or thrombocytopenia (*p* = 0.1672). Patients receiving combination therapy were more likely to be anemic at the end of therapy (odds ratio [OR] = 2.333, 95% confidence interval [CI] 0.999, 5.446). Similarly, patients receiving longer durations of nafcillin were more likely to experience anemia (OR 1.774, 95% CI 1.382, 2.370) and neutropenia (OR 1.256, 95% CI 1.024, 1.540). The use of nafcillin does not significantly affect myelosuppression in pediatric patients, although longer durations of therapy may result in increased rates of neutropenia and anemia. Combination therapy with rifampin may result in increased rates of neutropenia.

## 1. Introduction

Nafcillin is a beta-lactam antibiotic utilized primarily to treat infections caused by methicillin-sensitive *Staphylococcus aureus* [1]. Rifampin is an inhibitor of RNA polymerase and can be utilized as adjunct treatment with nafcillin for synergy in staphylococcal infections [2]. Staphylococcal infections can be complicated by bacterial biofilm production. The synergistic effect of rifampin provides greater antibacterial activity and broadens the spectrum of anti-staphylococcal activities [3]. Rifampin is not utilized as monotherapy due to the high incidence of resistance as an individual agent and inability to effectively penetrate gram-positive organisms when used alone [2].

Myelosuppression is a rarely documented, but potentially serious, adverse reaction of both nafcillin and rifampin. Current literature has only assessed the incidence of myelosuppression in patients receiving nafcillin or rifampin monotherapy. Two pediatric studies and one adult study demonstrated the potential for myelosuppression with nafcillin monotherapy. One retrospective study that included 58 patients receiving nafcillin reported neutropenia in 17.2%, which was not significantly different from other antibiotics studied [4]. Another study of pediatric patients found that 3.7% had bone marrow suppression, but there was no comparative group [5]. A study in adults reported higher rates of neutropenia with nafcillin (8.4%) compared with cefazolin (3.3%), but this difference was not statistically significant [6].

There are even fewer reports assessing myelosuppressive outcomes of rifampin use. A few case reports describe thrombocytopenia and hemolytic anemia following the use of rifampin; only one observational study in pediatric patients demonstrated thrombocytopenia occurring in infants at a rate of 121 per 1000 infant days [7,8,9]. There is currently no literature available regarding the effects of combination use of nafcillin and rifampin on bone marrow suppression. This study aims to address this literature gap and evaluate all components of myelosuppression (i.e., neutropenia, thrombocytopenia, and anemia). The purpose of this study is to describe the rates of myelosuppression in pediatric patients receiving nafcillin or a combination of nafcillin and rifampin therapy.

## 2. Materials and Methods

This single-center study was an Institutional Review Board (IRB)-approved retrospective chart review at a large academic children’s hospital. Individual medication use reports were generated for patients receiving nafcillin and rifampin from January 2014 to October 2020. Patients were eligible for inclusion in the study if they were aged 29 days through 17 years and received nafcillin with or without concomitant rifampin for a minimum of 72 h. Patients were excluded if they were actively or chronically immunosuppressed or were concomitantly using any medication contraindicated with the use of rifampin.

The primary endpoint of this study was to evaluate the incidence of myelosuppression as a composite endpoint of thrombocytopenia, anemia, and neutropenia in pediatric patients receiving nafcillin monotherapy or nafcillin and rifampin combination therapy. Secondary endpoints included assessing the incidence of thrombocytopenia, anemia, and neutropenia individually. The criteria for neutropenia were a white blood cell count of less than 4 × 10^9^/L or an absolute neutrophil count of less than 1.5 × 10^9^/L; the criteria for thrombocytopenia was a platelet count of less than 150 × 10^9^/L; the criteria for anemia were a hemoglobin of less than 12 g/dL or a hematocrit of less than 35%. These data points were collected via phlebotomy both prior to and after the initiation of therapy.

Data collection included patient demographics, medication order details, hematologic laboratory values and microbiological data. Descriptive statistics were utilized to analyze primary and secondary endpoints. Chi-squared tests and t-tests were utilized to assess the difference between the nafcillin monotherapy and combination groups for categorial and continuous variables, respectively. Associations between myelosuppression and other patient factors were explored via regression analysis.

## 3. Results

Of the 199 patients who received nafcillin or a combination of nafcillin and rifampin, 98 patients received nafcillin alone. The majority of patients were male with a mean age of 7.42 years (standard deviation [SD] 6.49) (Table 1). Patients receiving combination therapy weighed significantly and received a longer duration of nafcillin in the inpatient setting as well as a higher total weight-adjusted daily dose of nafcillin. Additional patient demographic data can be found in Table 1.

Patients receiving nafcillin were not significantly more likely to develop myelosuppression or any of the composite factors (neutropenia, thrombocytopenia, or anemia) (Table 2). However, patients receiving combination therapy were significantly more likely to be neutropenic after completion of combination therapy (*p* = 0.0003).

The addition of rifampin to therapy did not have a statistically significant effect on the development of myelosuppression (*p* = 0.0763). Similarly, the use of combination therapy did not have a statistically significant effect on the patient’s development of neutropenia (*p* = 0.2764) or thrombocytopenia (*p* = 0.1672). However, patients receiving combination therapy were significantly more likely to be classified as anemic at the end of therapy (OR = 2.333, 95% CI 0.999, 5.446, *p* = 0.0502). Similarly, patients receiving longer durations of therapy were more likely to experience anemia (OR 1.774, 95% CI 1.382, 2.370, *p* = 0.0001) and neutropenia (OR 1.256, 95% CI 1.024, 1.540, *p* = 0.0288).

## 4. Discussion

This study adds to the body of literature showing that perhaps the myelosuppression effects of nafcillin are not as great as previously thought. While this study is limited by the large number of patients who were myelosuppressed, more specifically anemic, prior to the initiation of nafcillin, the results still show that most patients did not develop any type of myelosuppression while receiving nafcillin alone. However, combination therapy with rifampin did result in an increased development of neutropenia, though it also showed a decreased incidence of thrombocytopenia. The latter may be explained by a clinical improvement due to appropriate antibiotic therapy and therefore a resolution of the low platelet count due to infection, or could be due to other factors not addressed by this study. The former finding is supported by case reports outlining similar neutropenia in pediatric patients [10,11].

This study did not aim to establish a clinical correlation of the potential for myelosuppression with the use of these medications, but this would be an excellent topic for future study. There are limited studies examining the clinical effects of the myelosuppression experienced with the use of nafcillin or nafcillin in combination with rifampin. Despite these hematologic changes, if the patient has a recovery of blood counts shortly after therapy is completed, concerns regarding these hematologic changes may be minimal in most patients.

The results of this study are limited by the potential for patients to be receiving multiple medications which may lead to the development of myelosuppression. Without a means to quantify the potential effect of each medication on the propensity of a patient to develop myelosuppression, it is challenging to account for the concurrent use of other such medications. Another limitation of this study is the variability in the timing of blood draws; some patients had their hematologic markers checked frequently, whereas others may only have had counts checked once during therapy. Another limitation is the differences in the demographics between the two patient groups at baseline, making it difficult to determine if the differences in outcomes could be due to underlying patient factors.

## 5. Conclusions

The use of nafcillin does not have a significant impact on myelosuppression, though longer durations of therapy may result in increased rates of neutropenia and anemia. Combination therapy with rifampin may result in increased rates of neutropenia. Further study is warranted to corroborate these results and to assess the clinical impact of these findings.

## Figures and Tables

**Table 1:** Baseline demographics.

Variable	Nafcillin	Nafcillin and Rifampin	*p* Value
Patients, *n* (%)	98 (49)	101 (51)	
Male sex, *n* (%)	60 (61)	64 (63)	
Weight, mean (SD), kg	20.93 (24.83)	30.46 (26.31)	0.016 *
Age, mean (SD), years	7.87 (7.39)	6.98 (5.49)	0.335
**Antibiotic Indication, *n***			
Respiratory	51	12	0.003 *
Bacteremia	21	28	0.000 *
Central nervous system	8	12	0.018 *
Skin and soft tissue infection	8	12	0.000 *
Osteomyelitis	2	22	0.018 *
Other	12	18	0.270
Length of stay, median (interquartile range, IQR), days	24.42 (10.19, 90.08)	15.64 (9.12, 23.89)	
Nafcillin duration of therapy, mean (SD), days	8.91 (7.87)	33.32 (14.67)	0.000 *
Rifampin duration of therapy, mean (SD), days		35.53 (16.22)	
Nafcillin total daily dose, mean (SD), mg/kg/day	132.87 (44.42)	159.11 (40.77)	0.000 *

* Significant at an alpha of <0.05.

**Table 2:** Primary and secondary endpoints.

Variable	Nafcillin	*p* Value	Nafcillin and Rifampin	*p* Value
**Myelosuppression, *n* (%)**				
Prior to therapy	64 (66.67)	0.2513	83 (82.18)	0.6547
After therapy	59 (61.46)		85 (84.16)	
**Neutropenia, *n* (%)**				
Prior to therapy	9 (9.38)	0.7630	4 (3.96)	0.0003 *
After therapy	10 (10.42)		20 (19.80)	
**Thrombocytopenia, *n* (%)**				
Prior to therapy	13 (13.68)	0.1317	18 (17.82)	0.0002 *
After therapy	8 (8.42)		4 (3.96)	
**Anemia, *n* (%)**				
Prior to therapy	56 (58.95)	0.8273	79 (78.22)	0.3938
After therapy	55 (57.89)		83 (82.18)	

* Significant at an alpha of <0.05.

## Data Availability

Not applicable.

## References

[1] Pediatric and Neonatal Lexi-Drugs/Nafcillin. Lexicomp App. UpToDate Inc.: Waltham, MA, USA. https://online.lexi.com/.

[2] Ma H., Cheng J., Peng L., Gao Y., Zhang G., Luo Z. (2020). Adjunctive rifampin for the treatment of staphylococcus aureus bacteremia with deep infections: A meta-analysis. PLoS ONE.

[3] Smith P.B., Cotton C.M., Hudak M.L., Sullivan J.E., Poindexter B.B., Cohen-Wolkowiez M., Boakye-Agyeman F., Lewandowski A., Anand R., Benjamin D.K. (2019). Rifampin pharmacokinetics and safety in preterm and term infants. Antimicrob. Agents Chemother..

[4] Maraqa N.F., Gomez M.M., Rathore M.H., Alvarez A.M. (2002). Higher occurrence of hepatotoxicity and rash in patients treated with oxacillin, compared with those treated with nafcillin and other commonly used antimicrobials. Clin. Infect. Dis..

[5] Benefield R.J., Barker B.C., Gast C.M., Alexander D.P. (2016). Patient variables associated with nafcillin plasma concentrations and toxicity. Pharmacotherapy.

[6] Youngster I., Shenoy E.S., Hooper D.C., Nelson S.B. (2014). Comparative evaluation of the tolerability of cefazolin and nafcillin for treatment of methicillin-susceptible staphylococcus aureus infections in the outpatient setting. Clin. Infect. Dis..

[7] Arnold C.J., Ericson J., Kohman J., Corey K.L., Oh M., Onabanjo J., Hornik C.P., Clark R.H., Benjamin D.K., Smith P.B. (2015). Rifampin use and safety in hospitalized infants. Am. J. Perinatol..

[8] Pau A.K., Fisher M.A. (1987). Severe thrombocytopenia associated with once-daily rifampin therapy. Drug Intell. Clin. Pharm..

[9] Ko C., Kwon K.C., Park J.W., Koo S.H., Shin S.Y. (2007). A case of rifampin induced hemolytic anemia combined with thrombocytopenia and acute renal failure. Korean J. Blood Transfus..

[10] Green G.R., Cohen E. (1978). Nafcillin-induced neutropenia in children. Pediatrics.

[11] Dutro M.P., Piecoro J.J., Wilson H.D. (1981). Nafcillin-induced neutropenia in two children. Am. J. Hosp. Pharm..

